# New Circumpapillary Retinal Nerve Fiber Layer Thickness and Bruch's Membrane Opening-Minimum Rim Width Assessment in Nonglaucomatous Eyes with Large Discs

**DOI:** 10.1155/2019/3431217

**Published:** 2019-10-23

**Authors:** Serife Bayraktar, Gulnar Sultanova, Zafer Cebeci, Emre Altinkurt, Belgin Izgi

**Affiliations:** Istanbul University, Istanbul Faculty of Medicine, Department of Ophthalmology, Istanbul, Turkey

## Abstract

**Purpose:**

To compare the new spectral-domain optical coherence tomography (SD-OCT) algorithm for measuring circumpapillary retinal nerve fiber layer (RNFL) thickness centered on Bruch's membrane opening (BMO), RNFL_BMO1_, with the conventional circumpapillary RNFL thickness measurement centered on the optic disc (RNFL_Dİ_), and assess the BMO-minimum rim width (BMO-MRW) in nonglaucomatous eyes with large discs.

**Methods:**

This prospective, cross-sectional, observational study included a total of 91 eyes of 91 patients having nonglaucomatous eyes with large discs (Group 1) and 50 eyes of 50 healthy subjects (Group 2). The optic nerve head (ONH) parameters obtained by confocal scanning laser ophthalmoscopy (CSLO), peripapillary RNFL thickness, BMO area, and BMO-MRW were imaged with SD-OCT.

**Results:**

The mean disc size was 3.06 ± 0.42 mm^2^ (range, 2.61–4.68) in Group 1 and 1.95 ± 0.23 mm^2^ (range, 1.6–2.43) in Group 2 (*p*=0.0001). The mean BMO area was 2.9 ± 0.58 mm^2^ (range, 1.26–4.62) in Group 1 and 2.05 ± 0.31 mm^2^ (range, 1.51–2.82) in Group 2 (*p*=0.0001). The difference between RNFL_Dİ and_ RNFL_BMO1_ measurements in Group 1 was stronger than in Group 2 because it was significant in all sectors in large discs. The mean global BMO-MRW thickness was significantly thinner in large discs; it was 252.95 ± 42.16 *µ* (range, 170–420) in Group 1 and 326.06 ± 73.39 *µ* (range, 210–440) in Group 2 (*p*=0.0001). There was a positive correlation between BMO-MRW thickness measurements and RNFL thickness parameters, both with RNFL_Dİ_ and RNFL_BMO1,_ in global and all optic nerve sectors except temporal quadrants with *r* = 0.257–0.431 (*p* ≤ 0.001–0.01) in Group 1. But in control group, Group 2, there was a weak correlation or no correlation between BMO-MRW thickness measurements and RNFL thickness parameters with *r* = −0.256–0.328 (*p*=0.797–0.02).

**Conclusion:**

The new circumpapillary RNFL scanning algorithm centered on BMO is better to assess the RNFL thickness and BMO-MRW in large discs for the early diagnosis of glaucoma.

## 1. Introduction

Glaucoma is a multifactorial, progressive optic neuropathy with characteristic visual field defects, abnormal thinning of the retinal nerve fiber layer (RNFL), and nonphysiological, characteristic cupping of the optic nerve head (ONH), which may result in vision loss and irreversible blindness. Structural and functional changes result from loss of retinal ganglion cells (RGCs) and their axons [1–4].

Detection of structural loss is fundamental in the diagnosis and management of glaucoma, and optical coherence tomography (OCT) is a commonly used imaging technology that can provide objective and reliable information on glaucomatous optic nerve damage by evaluating circumpapillary RNFL thickness [4–7]. In conventional spectral-domain OCT (SD-OCT), the operator manually positions the scan on the ONH. However, the disc margin can be challenging, and it varies among observers [8]. This may result in inaccurate RNFL thickness measurements. Recently, it was suggested that the Bruch's membrane opening-minimum rim width (BMO-MRW) is an anatomically and geometrically more accurate neuroretinal rim parameter that consists of the minimum distance between the BMO and the internal limiting membrane [9–13]. Glaucoma Module Premium Edition (GMPE), a software program that was recently introduced for the Spectralis SD-OCT (Spectralis, Heidelberg Engineering, Heidelberg, Germany), is based on this concept. It detects the BMO, and it measures the circumpapillary RNFL thickness by focusing on BMO. Moreover, the acquired BMO-MRW data are regionalized relative to the axis between the BMO and the fovea BMO (FoBMO) in each individual eye.

In glaucomatous eyes, the optic disc cup area is increased. Consequently, the cup-to-disc ratio is used to diagnose glaucoma. Although the number of ganglion axons in large-sized discs is not different from the number in average-sized discs, the cup area is also larger and the neuroretinal rim seems thinner in large discs, so it is important to differentiate whether or not the eye is glaucomatous [14–16]. Additionally, it is difficult to center the OCT scan circle in large discs to obtain a precise circumpapillary RNFL thickness measurement.

The present study aimed to evaluate circumpapillary RNFL thickness measurements based on BMO and compare the finding with conventional measurements in nonglaucomatous eyes with large discs. Hence, we evaluated the relationship between disc size and these parameters. We also assessed the BMO-MRW measurements and the angle of FoBMO in these eyes.

## 2. Materials and Methods

This prospective, cross-sectional, observational study included a total of 91 eyes of 91 patients with nonglaucomatous eyes with large discs (Group 1) and 50 eyes of 50 healthy subjects (Group 2). The study subjects were patients at the glaucoma unit in the Department of Ophthalmology at the Istanbul Faculty of Medicine from January 2017 to December 2017. The study was approved by the Ethics Committee of the Istanbul Faculty of Medicine, and all of the patients gave their informed consent. All investigations were conducted in accordance with the Declaration of Helsinki.

Each patient was subjected to a detailed ophthalmic assessment, including review of their medical history, measurement of their best-corrected visual acuity (BCVA) using a Snellen scale, ranging from 0.1 to 1.0, using slit-lamp biomicroscopy, Goldmann applanation tonometry, gonioscopy with a Goldmann 3-mirror lens, indirect dilated ophthalmoscopy, measurement of the central corneal thickness (CCT) (Ocuscan® RxP Ultrasound Pachymeter, Alcon, Inc., Irvine, CA, USA) and achromatic automated perimetry, using the 30-2 Swedish interactive threshold algorithm (SITA) standard program (Humphrey Visual Field Analyzer; Carl Zeiss-Meditec, Inc., Dublin, CA, USA), and confocal scanning laser ophthalmoscopy (CSLO) measurement (Heidelberg Retina Tomograph 3 (HRT3), Heidelberg Engineering GmbH, Heidelberg, Germany). The CSLO and SD-OCT measurements were performed on the same day.

Inclusion criteria for Group 1 were having a large disc of at least 2.45 mm^2^ in CSLO, a BCVA of 20/40 or better, refractive error within ±4.0D sphere, and a ±2.0 D cylinder with a clear cornea, clear ocular media, and a normal visual field. Exclusion criteria were diagnosis of glaucoma and narrow angle, optic disc abnormalities, such as tilted disc, optic neuropathies, advanced lens or corneal opacities, prior ocular surgery or laser treatment (except uncomplicated phacoemulsification surgery), intraocular diseases or ocular trauma, and coexisting neurological diseases affecting visual function or visual field. If both eyes fulfilled all the inclusion criteria and did not meet any of the exclusion criteria, the eye with the larger disc size in CSLO was selected.

The inclusion criteria for the control group (Group 2) were eyes with an average disc size (ranging between 1.63 and2.45 mm^2^ in CSLO), a BCVA of 20/20, a baseline intraocular pressure (IOP) <21 mmHg without any medication, a normal-appearing optic disc, normal RNFL thickness measurement and normal visual field, no ocular pathology or ocular trauma, and no family history of glaucoma. If both eyes of the patients satisfied the entry criteria, one eye of each subject was randomly selected for the study.

### 2.1. Imaging

**Figure 1:**
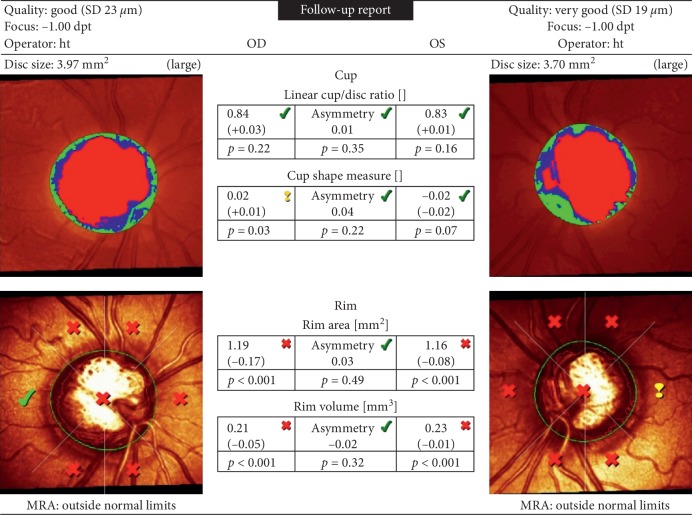
CSLO image of the optic discs of a patient in Group 1. Disc size of the right eye is larger, and the right eye was included in the study.

CSLO was performed by an experienced technician using the HRT3. A 3-dimensional (3D) topographic image, ranging from 384 × 384 × 16 to 384 × 384 × 64 pixels, was constructed from multiple focal planes axially along the ONH. The mean topography and reflectance images were automatically computed by the HRT3 software from three consecutive scans, centered on the ONH. We determined the large disc group by identifying eyes having a disc size of at least 2.45 mm^2^ in the HRT3 (Figure 1). This threshold was set according to the parameters indicated in the CSLO V.3.2.0.0 software. We defined mean pixel height standard deviation >30 mm, decentration of images, underillumination, and moving artifacts as exclusion parameters for image quality.

**Figure 2:**
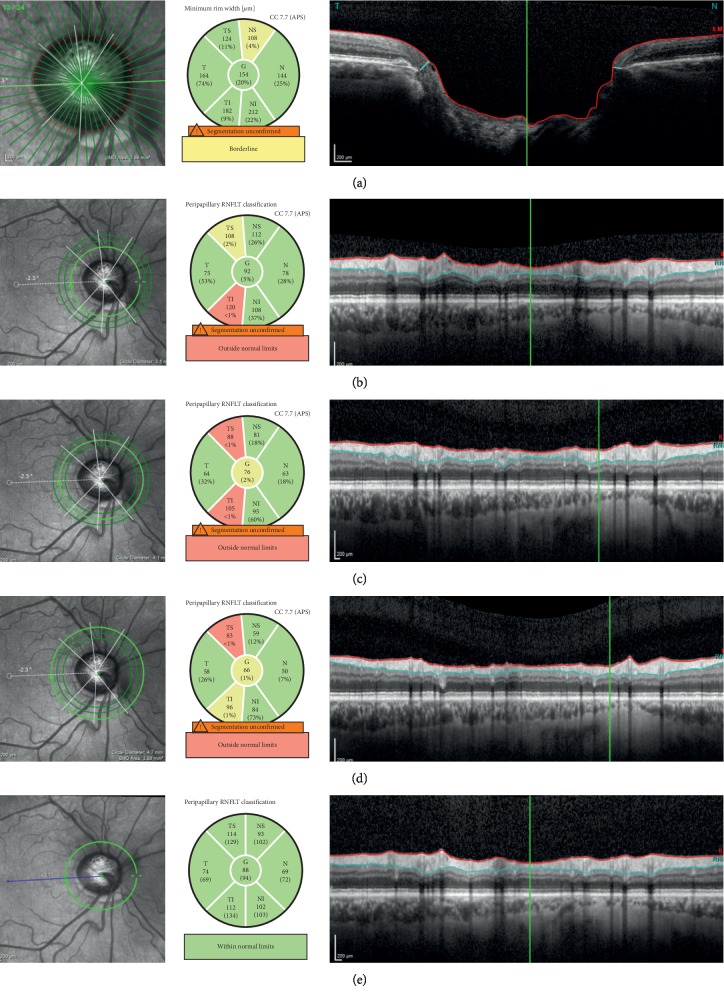
Measurements of the circumpapillary RNFL thickness (between red and blue line) using the new method (RNFL_BMO_) (a–d) and the conventional method (RNFL_Dİ_), FoBMO(°) angles (dashed white line) (e). (a) Determination of the center of BMO (red dots), infrared image indicating 24 locations where the radial B-scan images (dark green radial lines) were obtained and BMO-MRW thickness (blue arrow) measurements by centering on BMO. (b) RNFL_BMO1_, peripapillery circle with a diameter of 3.5 mm. (c) RNFL_BMO2_ peripapillary circle with a diameter of 4.1 mm. (d) RNFL_BMO3_ peripapillery circle with a diameter of 4.7 mm. (e) Conventional RNFL_Dİ_ measurement using the scan circle manually located by the examiner and FoDisc(°) angle (blue line).

The peripapillary area was imaged using Spectralis SD-OCT. Using the conventional mode, the operator first centered the circular scan on the optic disc (RNFL_DI_) and then focused on BMO (RNFL_BMO_; Figure 2). The software of the SD-OCT device provides a global average RNFL thickness and a mean RNFL thickness for each of the six sectors relative to the foveal disc (FoDisc) axis as follows: nasal superior (NS, 90–135°), nasal (N, 135–225°), nasal inferior (NI, 225–270°), temporal inferior (TI, 270–315°), temporal (T, 315–45°), and temporal superior (TS, 45–90°).

The RNFL_BMO_ measurements were taken using the new software (GMPE) in which circular scan images are centered on BMO. In this mode, the OCT device automatically detects BMO in 24 high-resolution, 158 radial scans of the ONH, each averaged from 20 to 30 individual B-scans, with 1536 A-scans per B-scan acquired with a scanning speed of 40,000 A-scans/second. Defining the anatomic map before image acquisition and use of the anatomic positioning system ensures that OCT images were acquired at fixed and known retinal locations relative to the fovea and the BMO center, which serve as anatomic landmarks for each individual eye. Then, three circular scans along the peripapillary circles, with diameters of 3.5 mm, 4.1 mm, and 4.7 mm, measured three sets of circumpapillary RNFL thicknesses centered on the BMO (RNFL_BMO1_, RNFL_BMO2_, and RNFL_BMO3_, respectively). Each scan circle produces a global average, and the mean thickness for each of the six sectors relative to the foveal BMO (FoBMO) axis is as follows: NS (85–125°), N (125–235°), NI (235–275°), TI (275–315°), T (315–45°), and TS (45–85°) [12, 17].

The FoDisc and FoBMO axes were obtained automatically when the RNFL_DI_ and RNFL_BMO_ scanning occurred, respectively. The FoDisc (or FoBMO) axis was defined as the angle between the fovea and the optic disc (or BMO) center relative to the horizontal axis of the image-acquisition frame.

Well-centered scans with correct retinal segmentation and quality score >20 were accepted.

### 2.2. Statistical Analysis

All statistical analyses were performed using SPSS software (SPSS for Windows version 23.0; SPSS Inc., Chicago, IL, USA). In addition to descriptive statistical methods (mean, standard deviation, and frequency, percentage), the Kolmogorov–Smirnov nonparametric test was used to evaluate the normal distribution of numerical data. Student's *t*-test was used to compare the quantitative data, if two independent groups with parametric test assumptions were provided. Analysis of variance (ANOVA) was used to compare more than two independent groups. To analyze the differences after conducting ANOVA, Tukey's test was used if the variances were found to be equal; if not, the Tamhane test was used. Because the parametric test assumptions were not found, the Mann–Whitney *U* test was used to compare the quantitative data of two independent groups, and the Kruskal–Wallis test was used to compare that data for more than two groups. Pearson's correlation analysis was used to determine the relationship between the measurement variables.

## 3. Results

A total of 141 eyes of 141 patients were enrolled in this study; 91 (64.5%) eyes of 91 patients with nonglaucomatous eyes with large discs were assigned to Group 1 and 50 (35.5%) eyes of 50 patients were assigned to the control group (Group 2). Ocular hypertension (OHT) was also present in 30 (21.3%) of the eyes with large discs (Group 1).

The epidemiologic characteristics and baseline data of the included eyes are shown in Table 1. The differences between gender (*p*=0.03), BCVA (*p*=0.015), IOP (*p*=0.109), and CCT (*p*=0.487) were not statistically significant. Significant differences were found for age (*p*=0.0001), disc size in CSLO (*p*=0.0001), linear *c/d* ratio in CSLO (*p*=0.0001), rim area in CSLO (*p*=0.004), and BMO area in SD-OCT (*p*=0.0001).

The SD-OCT-based RNFL thickness parameters in both groups, including the global and six optic nerve sectors by centering on the optic nerve (RNFL_Dİ_), are shown in Table 2. The difference was statistically significant in the global and in all quadrants except the nasal superior quadrant (*p*=0.0001, *p*=0.012,  *p*=0.413, *p*=0.038, *p*=0.006, *p*=0.023,  and *p*=0.019 in the global, temporal superior, nasal superior, nasal, nasal inferior, temporal inferior, and temporal quadrants, respectively).

The SD-OCT-based RNFL thickness parameters, including the global and six optic nerve sectors by centering on BMO (RNFL_BMO1_) at 3.5 mm, using the GMPE module in both groups, are shown in Table 3. The difference was statistically significant in the global and in all quadrants, except the nasal superior and temporal inferior quadrants (*p*=0.0001,  *p*=0.012,  *p*=0.073,  *p*=0.007,  *p*=0.012,  *p*=0.2,  and *p*=0.014 in the global, temporal superior, nasal superior, nasal, nasal inferior, temporal inferior, and temporal quadrants, respectively).

Comparison of the RNFL thickness parameters, including six optic nerve sectors by centering on the optic nerve (RNFL_Dİ_) and BMO (RNFL_BMO1_), is shown in Table 4. In Group 1, there were significant differences between the RNFL_Dİ_ and RNFL_BMO1_ thickness measurements in the global and in all the sectors (*p* ≤ 0.001,  *p*=0.036,  *p*=0.002,  *p* ≤ 0.001,  *p*=0.016,  *p* ≤ 0.001,  and *p*=0.014 in the global, temporal superior, nasal superior, nasal, nasal inferior, temporal inferior, and temporal quadrants, respectively). In Group 2, there were significant differences in the global, nasal superior, nasal, and temporal inferior quadrants (*p* ≤ 0.001 in all). However, in the temporal superior, nasal inferior, and temporal quadrants (*p*=0.418,  *p*=0.068,  and *p*=0.065, respectively), the difference was not statistically significant. The difference between the RNFL_Dİ and_ RNFL_BMO1_ measurements was greater in Group 1 than in Group 2.

The BMO-MRW thickness measurements by centering on BMO, using the GMPE module, including six optic nerve sectors in the two groups, are shown in Table 5. The differences between the two groups were statistically significant in the global and all the sectors (*p*=0.0001 in all). The BMO-MRW thicknesses seemed to be thinner in all optic nerve sectors in eyes with large discs.

The correlation between the BMO-MRW thickness measurements by centering on BMO, using the GMPE module and the RNFL thickness parameters, including six optic nerve sectors by centering on the optic nerve (RNFL_Dİ_) and BMO (RNFL_BMO1_) in the two groups, is shown in Table 6. A positive correlation was found between the BMO-MRW thickness measurements and the RNFL thickness parameters—for both RNFL_Dİ_ and RNFL_BMO1_—in the global and all the optic nerve sectors, except the temporal quadrants, with *r* = 0.257–0.431 (*p* ≤ 0.001–0.01) in Group 1. However, in the control group (Group 2), a weak correlation or no correlation was found between the BMO-MRW thickness measurements and the RNFL thickness parameters, with *r* = −0.256–0.328 (*p*=0.797–0.02).

The correlation of disc size obtained by CSLO with the global SD-OCT and the CSLO parameters is shown in Table 7. No correlation was found between disc size and the linear *c/d* ratio in Group 1, with *r* = 0.052 (*p*=0.622); however, the correlation was strong in Group 2, with *r* = 0.409 (*p*=0.003). A positive and similar correlation was found between disc size and rim area in both groups, with *r* = 0.371 (*p* ≤ 0.001) and *r* = 0.386 (*p*=0.006). The correlation between disc size and BMO area was stronger in Group 1 (*r* = 0.602, *p* ≤ 0.001) than in Group 2 (*r* = 0.454, *p*=0.001). No correlations were found between disc size and the global BMO-MRW thickness measurements and the RNFL thickness parameters.

The correlations between the rim area in CSLO and the global SD-OCT and CSLO parameters are shown in Table 8. A negative strong correlation was found between the rim area and the linear *c/d* ratio in both groups; however, the correlation was stronger in Group 1, with *r* = −0.860 (*p* ≤ 0.001) and *r* = −0.626 (*p* ≤ 0.001), respectively. Rim area was found to be strongly correlated with global BMO-MRW thickness measurements in Group 1, with *r* = 0.593 (*p* ≤ 0.001); in Group 2, no correlation was found, with *r* = 0.025 (p = 0.863). No correlations were found between the rim area and the BMO area and RNFL thickness parameters.

The FoDisc and FoBMO angles in SD-OCT are shown in Table 9. No significant difference in FoDisc and FoBMO was observed between the two groups (*p*=0.249 and *p*=0.059). Moreover, a comparison of the FoDisc and FoBMO within the two groups was not statistically significant (*p*=0.105,  *p*=0.623).

## 4. Discussion

The assessment of circumpapillary RNFL thickness is accepted to be essential in the diagnosis and follow-up of glaucoma. SD-OCT is one of the imaging modalities that is most often used worldwide to evaluate ONH and the neuroretinal rim. Recently, BMO-based SD-OCT of the optic disc has become a major clinical aid in glaucoma detection.

It is challenging to recognize the morphological changes in large discs and to detect whether or not the eye is glaucomatous because a large disc area is significantly correlated with the optic cup area in both glaucomatous and nonglaucomatous eyes [14–17]. The present study investigated circumpapillary RNFL thickness measurements based on BMO and compared the findings with conventional measurements in nonglaucomatous eyes with large discs using the new GMPE software for SD-OCT. The BMO-MRW measurements and the angle of FoBMO in these eyes were also assessed.

Several previous studies have discussed the relationship between disc size and RNFL thicknesses [15, 18–23]. Onmez et al. [15] evaluated and compared RNFLT measurements between large and normal-sized discs using Stratus OCT; they found that the RNFL thicknesses were similar in both study groups. They also reported a weak correlation between RNFL thickness and optic disc size. In contrast, Gür Güngör et al. [18] compared the measurements of RNFL thicknesses in three different ONH size groups using Cirrus SD-OCT. They reported significant differences for superior, inferior, and average RNFL thickness between the ONH size groups, and they observed that the RNFL thicknesses in all quadrants increased with ONH size. Öztürker et al. [19] aimed to evaluate the optic disc and macular characteristics of eyes with macrodiscs using SD-OCT. They found no correlation between the average total, superior, or inferior pRNFL and ONH size.

Savini et al. [20] showed that RNFLT measurements obtained using Stratus OCT are positively correlated with ONH size. They found that the correlation may be the result of either an increased number of nerve fibers in eyes with larger discs or a smaller distance between the circular scan and the true ONH margin.

Kaushik et al. [21] scanned the peripapillary RNFL of 32 normal eyes with the fast-scanning protocol at a diameter of 3.4 mm using Stratus OCT; they found that the disc area did not affect the RNFL thickness measurement. They suggested that RNFL thickness is dependent on the distance from the center of the optic disc rather than the point of exit from the scleral canal and that RNFL thickness should be measured at similar distances from the center of the optic disc, regardless of the size of the scleral canal.

Mansoori et al. [22] investigated the influence of disc area on the RNFLT measurement using SD-OCT; they found that the mean and quadrant RNFLT did not show a significant correlation with disc area among the subjects in the subgroup of eyes with a disc area <3 mm^2^ and in the subgroup of eyes with a disc area ranging between 3-4 mm^2^. However, in the subgroup of eyes with a disc area >4 mm^2^, average RNFLT, and superior and temporal quadrant RNFLT showed a negative correlation with disc area, and the difference was statistically significant.

Savini et al. [23] measured RNFLT using a 3.4 mm diameter scan circle and two customized-diameter scans (at 0.5 mm and 1 mm from the ONH edge) with a Stratus OCT. They confirmed that the RNFLT measurements are affected by the ONH size. When a fixed-diameter circular scan is used, larger discs had higher values than smaller discs; conversely, when the diameter was adjusted on the basis of ONH size, the larger discs had lower values. They suggested that a normative database of peripapillary RNFLT should be created to correct ONH size.

In the present study, no correlation was found between disc size and the global RNFL thickness parameters when centering on the optic nerve (RNFL_Dİ_) or centering on the BMO (RNFL_BMO1_). The mean global RNFL_Dİ_ thickness was 96.99 ± 10.31 *µ* in Group 1 and 103.6 ± 7.03 *µ* in Group 2. Moreover, the mean global RNFL_BMO1_ was 99.6 ± 11.95 *µ* in Group 1 and 106.16 ± 6.93 *µ* in Group 2. This means that, in both the GMPE module and the conventional SD-OCT assessments, RNFL thicknesses were found to be thinner in nonglaucomatous large discs. Additionally, the difference between the RNFL thicknesses (RNFL_Dİ_ and RNFL_BMO1_ measurements) was greater in Group 1 than in Group 2.

Enders et al. [24] compared the margin-based rim area measurements from CSLT and the BMO-based measurements from SD-OCT in large discs. This study also aimed to create a reference for large ONHs in SD-OCT diagnostics. They found that BMO-MRW seems to be thinner in larger optic discs when the findings were compared with the normative data. In their study group, the mean global BMO-MRW thickness was 234.84 ± 48.3 *µ*. Similarly, in the present study, the mean global BMO-MRW thickness was 252.95 ± 42.16 *µ* in Group 1 and 326.06 ± 73.39 *µ* in Group 2. The BMO-MRW was significantly thinner in the larger optic discs, as seen in Table 5. Enders et al. [24] also found that BMO-MRW correlates better than the CSLT parameters with the RNFLT measured using SD-OCT. The correlation between BMO-MRW and global RNFLT was stronger than the correlation between the CSLT rim area and global RNFLT. Similarly, the present study found no correlation between the rim area and global RNFL_Dİ_ and RNFL_BMO1_ thickness measurements in Group 1 (*r* = 0.128, *p*=0.228 and *r* = 0.099, *p*=0.348, respectively) and in Group 2 (*r* = 0.069, *p*=0.634 and *r* = 0.199, *p*=0.165, respectively), as seen in Table 8. A correlation was found between BMO-MRW and the global RNFL_Dİ_ and RNFL_BMO1_ thickness parameters, especially in Group 1, as seen in Table 6 (*r* = 0.347, *p*=0.001 and *r* = 0.333, *p*=0.001, respectively).

Toshev et al. [25] compared the diagnostic performance and evaluated the diagnostic agreement of early glaucoma detection between CSLO and SD-OCT. They investigated 55 open-angle glaucoma patients and 42 eyes of 42 healthy controls. They showed that the BMO-MRW assessment with SD-OCT performed well in detecting glaucomatous damage (Spectralis global BMO-MRW AUROC = 0.956).

Enders et al. [26] assessed the diagnostic power of OCT to detect glaucoma in eyes with glaucomatous large discs. They also evaluated the structure-function relationship of OCT-based morphometric data along different classifications of the glaucomatous visual field. This study's cohort included 125 eyes of 125 patients with large discs (44 glaucoma, 11 ocular hypertension, and 70 healthy controls). They found that BMO-MRW had the best diagnostic power to discriminate glaucoma patients from normal controls in comparison with RNFLT and the rim area in CSLT. Moreover, BMO-MRW seemed to reflect the structure-function relationship better than the other two parameters.

In summary, the global RNFL and BMO-MRW are thinner in eyes with nonglaucomatous large discs. The difference between the RNFL_Dİ_ and RNFL_BMO1_ thicknesses is more significant in these types of eyes. The correlation between RNFLT and BMO-MRW is stronger in eyes with large discs in comparison with the healthy controls. As reported in the literature, BMO-MRW is very important in the early diagnosis of glaucoma. With reference to these results, it is better to assess RNFLT and BMO-MRW using the GMPE module of Spectralis SD-OCT in eyes with large discs for the early diagnosis of glaucoma. The normative values with large discs in the literature are limited, and the present study's data about 91 eyes of 91 patients are also significant. However, studies with a larger sample size with different groups, such as glaucoma and OHT with large discs, are needed.

## Figures and Tables

**Table 1:** Epidemiological and baseline data.

	Group 1	Group 2	*p*
*N*	91	50	

Gender, *n* (%)			**0.03**
Men	30 (33%)	13 (26%)	
Women	61 (67%)	37 (74%)	

Age (years)			0.0001^a^
Mean (SD)	53.21 ± 17.13	44.32 ± 9.65	
Range	15 to 87	23 to 63	

Eye, *n* (%)			
Right	47 (52%)	37 (74%)	
Left	44 (48%)	13 (26%)	

BCVA (Snellen)			0.015^b^
Mean (SD)	0.97 ± 0.1	1.0 ± 0	
Range	0.5 to 1.0	1.0 to 1.0	

IOP (mmHg)			0.109^b^
Mean (SD)	15.77 ± 3.09	14.80 ± 2.31	
Range	11 to 26	10 to 21	

CCT (*µ*)			0.487^a^
Mean (SD)	547.03 ± 37.25	551.56 ± 36.25	
Range	441 to 651	477 to 624	

Disc size in CSLO (mm^2^)			0.0001^b^
Mean (SD)	3.06 ± 0.42	1.95 ± 0.23	
Range	2.61 to 4.68	1.6 to 2.43	

Linear *c/d* ratio in CSLO (mm^2^)			
Mean (SD)	0.65 ± 0.13	0.46 ± 0.11	0.0001^b^
Range	0.02 to 0.94	0.01 to 0.68	

Rim area in CSLO (mm^2^)			
Mean (SD)	1.69 ± 0.48	1.5 ± 0.19	0.004^b^
Range	0.35 to 3.46	1.15 to 2.12	

BMO area in SD-OCT (mm^2^)			
Mean (SD)	2.9 ± 0.58	2.05 ± 0.31	0.0001^a^
Range	1.26 to 4.62	1.51 to 2.82	

Group 1: nonglaucomatous eyes with large discs. Group 2: controls. BCVA: best-corrected visual acuity; IOP: intraocular pressure; CCT: central corneal thickness; CSLO: confocal scanning laser ophthalmoscopy; *c/d* ratio: cup-to-disc ratio; BMO: Bruch's membrane opening; SD-OCT: spectral-domain optical coherence tomography. ^a^Student's *t*-test. ^b^Mann–Whitney *U* test.

**Table 2:** Retinal nerve fiber layer thickness measurements by centering on the optic disc (RNFL_Di_) using the conventional mode including six optic nerve sectors.

	RNFL_Di_	Number	Mean (SD) (*µ*)	Range (*µ*)	*p*
Group 1	Global	91	96.99 ± 10.31	72–118	**0.0001** ^**a**^
Group 2		50	103.6 ± 7.03	90–119	

Group 1	Temporal	91	133.49 ± 21.25	65–183	**0.012** ^**a**^
Group 2	superior	50	140.9 ± 13.11	117–170	

Group 1	Nasal	91	108.09 ± 21.70	66–195	0.413^a^
Group 2	superior	50	111.04 ± 17.77	72–165	

Group 1	Nasal	91	73.54 ± 14.22	44–123	**0.038** ^**a**^
Group 2		50	78.72 ± 13.65	56–114	

Group 1	Nasal	91	106.14 ± 23.03	44–173	**0.006** ^**a**^
Group 2	inferior	50	116.98 ± 20.62	82–171	

Group 1	Temporal	91	141.57 ± 20.34	94–182	**0.023** ^**a**^
Group 2	inferior	50	148.68 ± 15.77	120–206	

Group 1	Temporal	91	70.56 ± 12.39	36–99	**0.019** ^**b**^
Group 2		50	74.8 ± 9.83	58–101	

^a^Student's *t*-test. ^b^Mann–Whitney *U* test.

**Table 3:** Retinal nerve fiber layer thickness measurements by centering on BMO (RNFL_BMO1_) at 3.5 mm using GMPE module including six optic nerve sectors.

	RNFL_BMO1_	Number	Mean (SD) (*µ*)	Range (*µ*)	*p*
Group 1	Global	91	99.6 ± 11.95	72–149	**0.0001** ^**a**^
Group 2		50	106.16 ± 6.93	90–123	

Group 1	Temporal	91	129.91 ± 24.39	53–208	**0.012** ^**a**^
Group 2	superior	50	138.78 ± 16.9	105–178	

Group 1	Nasal	91	112.18 ± 24.99	70–189	0.073^b^
Group 2	superior	50	119.76 ± 22.18	83–200	

Group 1	Nasal	91	81.86 ± 13.89	44–119	**0.007** ^**a**^
Group 2		50	88.04 ± 10.98	72–110	

Group 1	Nasal	91	109.19 ± 25.95	22–164	**0.012** ^**a**^
Group 2	inferior	50	118.76 ± 18.17	87–164	

Group 1	Temporal	91	151.91 ± 19.37	101–202	**0.200** ^**a**^
Group 2	inferior	50	156.12 ± 16.95	129–210	

Group 1	Temporal	91	72.46 ± 11.06	52–102	**0.014** ^**b**^
Group 2		50	76.12 ± 8.15	61–96	

^a^Student's *t*-test. ^b^Mann–Whitney *U* test.

**Table 4:** Spectral-domain optical coherence tomography- (SD-OCT-) based peripapiller retinal nerve fiber layer (RNFL) thickness parameters including six optic nerve sectors by centering optic nerve (RNFL_Dİ_) and Bruch' membrane opening (RNFL_BMO1_).

	Global	Temporal superior	Nasal superior	Nasal	Nasal inferior	Temporal inferior	Temporal
Group 1							
RNFL_Di_ (*µ*)	96.99 ± 10.31	133.49 ± 21.25	108.09 ± 21.70	73.54 ± 14.22	106.14 ± 23.03	141.57 ± 20.34	70.56 ± 12.39
RNFL_BMO1_ (*µ*)	99.6 ± 11.95	129.91 ± 24.39	112.18 ± 24.99	81.86 ± 13.89	109.19 ± 25.95	151.91 ± 19.37	72.46 ± 11.06
*Z*^a^	−3.783^c^	−2.101^b^	−3.088^c^	−6.740^c^	−2.408^c^	−6.458^c^	−2.470^c^
*p*	**≤0.001**	**0.036**	**0.002**	**≤0.001**	**0.016**	**≤0.001**	**0.014**

Group 2							
RNFL_Di_ (*µ*)	103.6 ± 7.03	140.9 ± 13.11	111.04 ± 17.77	78.72 ± 13.65	116.98 ± 20.62	148.68 ± 15.77	74.8 ± 9.83
RNFL_BMO1_ (*µ*)	106.16 ± 9.93	138.78 ± 16.9	119.76 ± 22.18	88.04 ± 10.98	118.76 ± 18.17	156.12 ± 16.95	76.12 ± 8.15
*Z*^a^	−3.713^c^	−0.810^b^	−3.567^c^	−5.404^c^	−1.827^c^	−3.946^c^	−1.844^c^
*p*	**≤0.001**	0.418	**≤0.001**	**≤0.001**	0.068	**≤0.001**	0.065

^a^Wilcoxon signed rank test. ^b^based on positive ranks. ^c^based on negative ranks.

**Table 5:** BMO-MRW thickness measurements by centering on BMO by using GMPE module including six optic nerve sectors.

	BMO-MRW	Number	Mean (SD) (*µ*)	Range (*µ*)	*p*
Group 1	Global	91	252.95 ± 42.16	170–420	**0.0001** ^b^
Group 2		50	326.06 ± 73.39	210–440	

Group 1	Temporal	91	248.88 ± 47.90	143–400	**0.0001** ^a^
Group 2	superior	50	335.04 ± 52.64	257–490	

Group 1	Nasal	91	277.92 ± 57.09	175–520	**0.0001** ^a^
Group 2	superior	50	382.90 ± 57.97	291–575	

Group 1	Nasal	91	262.59 ± 60.22	114–531	**0.0001** ^a^
Group 2		50	370.98 ± 46.03	216–461	

Group 1	Nasal	91	307.16 ± 57.22	171–527	**0.0001** ^b^
Group 2	inferior	50	405.60 ± 48.96	299–503	

Group 1	Temporal	91	285.15 ± 46.22	183–492	**0.0001** ^b^
Group 2	inferior	50	369.06 ± 48.20	278–460	

Group 1	Temporal	91	193.54 ± 36.37	123–316	**0.0001** ^b^
Group 2		50	259.98 ± 41.67	182–359	

^a^Student's *t*-test. ^b^Mann–Whitney *U* test.

**Table 6:** The correlation between the BMO-MRW thickness measurements by centering on BMO, using the GMPE module and the RNFL thickness parameters, including six optic nerve sectors by centering on the optic nerve (RNFL_Dİ_) and BMO (RNFL_BMO1_).

		Global	Temporal superior	Nasal superior	Nasal	Nasal inferior	Temporal inferior	Temporal
Group 1	BMO-MRW (*µ*)	252.95 ± 42.16	248.88 ± 47.90	277.92 ± 57.09	262.59 ± 60.22	307.16 ± 57.22	285.15 ± 46.22	193.54 ± 36.37
	RNFL_Dİ_ (*µ*)	96.99 ± 10.31	133.49 ± 21.25	108.09 ± 21.70	73.54 ± 14.22	106.14 ± 23.03	141.57 ± 20.34	70.56 ± 12.39
	*p*	0.001	0.002	≤0.001	0.014	0.001	0.012	0.140
	Correlation (Pearson)	0.347^*∗∗*^	0.322^*∗∗*^	0.431^*∗∗*^	0.257^*∗*^	0.347^*∗∗*^	0.261^*∗*^	0.185
	BMO-MRW (*µ*)	252.95 ± 42.16	248.88 ± 47.90	277.92 ± 57.09	262.59 ± 60.22	307.16 ± 57.22	285.15 ± 46.22	193.54 ± 36.37
	RNFL_BMO1_ (*µ*)	99.6 ± 11.95	129.91 ± 24.39	112.18 ± 24.99	81.86 ± 13.89	109.19 ± 25.95	151.91 ± 19.37	72.46 ± 11.06
	*p*	0.001	0.001	≤0.001	0.003	0.013	0.003	0.696
	Correlation (Pearson)	0.333^*∗∗*^	0.333^*∗∗*^	0.388^*∗∗*^	0.306^*∗∗*^	0.259^*∗*^	0.312^*∗∗*^	0.042

Group 2	BMO-MRW (*µ*)	326.06 ± 73.39	335.04 ± 52.64	382.90 ± 57.97	370.98 ± 46.03	405.60 ± 48.96	369.06 ± 48.20	259.98 ± 41.67
	RNFL_Dİ_ (*µ*)	103.6 ± 7.03	140.9 ± 13.11	111.04 ± 17.77	78.72 ± 13.65	116.98 ± 20.62	148.68 ± 15.77	74.8 ± 9.83
	*p*	0.504	0.073	0.113	0.036	0.178	0.02	0.190
	Correlation (Pearson)	−0.097	−0.256	−0.227	0.297^*∗*^	0.194	0.328^*∗*^	−0.189
	BMO-MRW (*µ*)	326.06 ± 73.39	335.04 ± 52.64	382.90 ± 57.97	370.98 ± 46.03	405.60 ± 48.96	369.06 ± 48.20	259.98 ± 41.67
	RNFL_BMO1_ (*µ*)	106.16 ± 6.93	138.78 ± 16.9	119.76 ± 22.18	88.04 ± 10.98	118.76 ± 18.17	156.12 ± 16.95	76.12 ± 8.15
	*p*	0.708	0.790	0.759	0.130	0.175	0.104	0.797
	Correlation (Pearson)	0.054	0.039	0.045	0.217	0.195	0.233	−0.037

^*∗*^Correlation is significant at the 0.05 level (2-tailed). ^*∗∗*^Correlation is significant at the 0.01 level (2-tailed).

**Table 7:** Correlation of disc size in CSLO with global SD-OCT and CSLO parameters.

		Linear *c/d* ratio	Rim area (CSLO)	BMO-MRW (*µ*)	BMO area (SD-OCT)	RNFL_Dİ_ (*µ*) global	RNFL_BMO1_ (*µ*) global
Disc size							
Group 1 (*n* = 91)	*p*	0.622	**≤0.001**	0.531	**≤0.001**	0.609	0.669
	Correlation (Pearson)	0.052	0.371^*∗∗*^	−0.066	0.602^*∗∗*^	0.054	0.045

Group 2 (*n* = 50)	*p*	0.003	0.006	0.572	0.001	0.421	0.351
	Correlation (Pearson)	0.409^*∗∗*^	0.386^*∗∗*^	−0.082	0.454^*∗∗*^	0.116	0.135

^*∗*^Correlation is significant at the 0.05 level (2-tailed). ^*∗∗*^Correlation is significant at the 0.01 level (2-tailed).

**Table 8:** Correlation of rim area in CSLO with global SD-OCT and CSLO parameters.

		Linear *c/d* ratio	BMO-MRW (*µ*) global	BMO area (SD-OCT)	RNFL_Dİ_ (*µ*) global	RNFL_BMO1_ (*µ*) global
Rim area *(CSLO)*						
Group 1 (*n* = 91)	*p*	**≤0.001**	**≤0.001**	0.534	0.228	0.348
	Correlation (Pearson)	−0.860	0.593	0.066	0.128	0.099

Group 2 (*n* = 50)	*p*	**≤0.001**	0.863	0.335	0.634	0.165
	Correlation (Pearson)	−0.626	0.025	0.139	0.069	0.199

^*∗*^Correlation is significant at the 0.05 level (2-tailed). ^*∗∗*^Correlation is significant at the 0.01 level (2-tailed).

**Table 9:** FoDisc(°) and FoBMO(°) angles in SD-OCT.

	Group 1	Group 2	*p*
Number	91	50	
FoDisc angle (°)	−5.06 ± 4.98	−4.84 ± 3.26	0.249^d^
FoBMO angle (°)	−6.23 ± 3.84	−5.03 ± 2.96	0.059^b^
*Z* ^a^	−1.621^b^	−0.492^b^	
*p*	0.105	0.623	

^d^Wilcoxon signed-rank test. ^e^based on positive ranks. ^f^based on negative ranks.

## Data Availability

The data used to support the findings of this study are available from the corresponding author upon request.

## References

[1] Quigley H. A. (1993). Open-angle glaucoma. *New England Journal of Medicine*.

[2] Weinreb R. N., Khaw P. T. (2004). Primary open-angle glaucoma. *The Lancet*.

[3] Harwerth R. S., Wheat J. L., Fredette M. J., Anderson D. R. (2010). Linking structure and function in glaucoma. *Progress in Retinal and Eye Research*.

[4] Bussel I. I., Wollstein G., Schuman J. S. (2014). OCT for glaucoma diagnosis, screening and detection of glaucoma progression. *British Journal of Ophthalmology*.

[5] Wollstein G., Schmann J. S., Price L. L. (2005). Optical coherence tomography longitudinal evaluation of retinal nerve fiber layer thickness in glaucoma. *Archives of Ophthalmology*.

[6] Schuman J. S., Hee M. R., Puliafito C. A. (1995). Quantification of nerve fiber layer thickness in normal and glaucomatous eyes using optical coherence tomography. *Archives of Ophthalmology*.

[7] Leung C. K.-S., Cheung C. Y.-L., Weinreb R. N. (2009). Retinal nerve fiber layer imaging with spectral-domain optical coherence tomography: a variability and diagnostic performance study. *Ophthalmology*.

[8] Iester M., Mikelberg F. S., Courtright P. (2013). Interobserver variability of optic disk variables measured by confocal scanning laser tomography. *American Journal of Ophthalmology*.

[9] Lee E. J., Lee K. M., Kim H., Kim T.-W. (2016). Glaucoma diagnostic ability of the new circumpapillary retinal nerve fiber layer thickness analysis based on Bruch’s membrane opening. *Investigative Opthalmology & Visual Science*.

[10] Chauhan B. C., Burgoyne C. F. (2013). From clinical examination of the optic disc to clinical assessment of the optic nerve head: a paradigm change. *American Journal of Ophthalmology*.

[11] Reis A. S. C., Sharpe G. P., Yang H., Nicolela M. T., Burgoyne C. F., Chauhan B. C. (2012). Optic disc margin anatomy in patients with glaucoma and normal controls with spectral domain optical coherence tomography. *Ophthalmology*.

[12] Chauhan B. C., Danthurebandara V. M., Sharpe G. P. (2015). Bruch’s membrane opening- minimum rim width and retinal nerve fiber layer thickness in a normal white population: a multi-centre study. *Ophthalmology*.

[13] Reis A. S. C., O’Leary N., Yang H. (2012). Influence of clinically invisible, but optical coherence tomography detected, optic disc margin anatomy on neuroretinal rim evaluation clinically invisible optic disc margin anatomy. *Investigative Opthalmology & Visual Science*.

[14] Hoffman E. M., Zangwill L. M., Crowston J. G., Weinreb R. N. (2007). Optic disc size and glaucoma. *Survey of Ophthalmology*.

[15] Onmez F. E., Satana B., Altan C., Basarir B., Demirok A. (2014). A comparison of optic nerve head topographic measurements by Stratus OCT in patients with macrodiscs and normal-sized healthy discs. *Journal of Glaucoma*.

[16] Okimoto S., Yamashita K., Shibata T., Kiuchi Y. (2015). Morphological features and important parameters of large optic discs for diagnosing glaucoma. *PLoS One*.

[17] Danthurebandara V. M., Sharpe G. P., Hutchison D. M. (2015). Enhanced structure-function relationship in glaucoma with an anatomically and geometrically accurate neuroretinal rim measurement. *Investigative Ophthalmology & Visual Science*.

[18] Gür Güngör S., Akman A., Küçüködük A., Çolak M. (2016). Retinal nerve fiber layer thicknesses in three different optic nerve head size groups measured by Cirrus spectral domain optical coherence tomography. *Türk Oftalmoloji Dergisi*.

[19] Öztürker Z. K., Eltutar K., Karini B., Erkul S. Ö., Osmanbaşoğlu Ö. A., Sultan P. (2016). Optic nerve head topography and retinal structural changes in eyes with macrodisks: a comparative study with spectral domain optical coherence tomography. *Clinical Ophthalmology*.

[20] Savini G., Zanini M., Carelli V., Sadun A. A., Ross-Cisneros F. N., Barboni P. (2005). Correlation between retinal nerve fibre layer thickness and optic nerve head size: an optical coherence tomography study. *British Journal of Ophthalmology*.

[21] Kaushik S., Pandav S. S., Ichhpujani P., Gupta A. (2009). Fixed-diameter scan protocol preferable for retinal nerve fibre layer measurement by optical coherence tomography in all sizes of optic discs. *British Journal of Ophthalmology*.

[22] Mansoori T., Balakrishna N., Viswanath K. (2014). Influence of disc area on retinal nerve fiber layer thickness measurement by spectral domain optical coherence tomography. *Indian Journal of Ophthalmology*.

[23] Savini G., Barboni P., Carbonelli M., Zanini M. (2007). The effect of scan diameter on retinal nerve fiber layer thickness measurement using Stratus optic coherence tomography. *Archives of Ophthalmology*.

[24] Enders P., Schaub F., Hermann M. M., Cursiefen C., Heindl L. M. (2017). Neuroretinal rim in non-glaucomatous large optic nerve heads: a comparison of confocal scanning laser tomography and spectral domain optical coherence tomography. *British Journal of Ophthalmology*.

[25] Toshev A. P., Lamparter J., Pfeiffer N., Hoffmann E. M. (2017). Bruch’s membrane opening-minimum rim width assessment with spectral-domain optical coherence tomography performs better than confocal scanning laser ophthalmoscopy in discriminating early glaucoma patients from control subjects. *Journal of Glaucoma*.

[26] Enders P., Schaub F., Adler W. (2018). Bruch’s membrane opening-based optical coherence tomography of the optic nerve head: a useful diagnostic tool to detect glaucoma in macrodiscs. *Eye*.

